# Pennsylvania State Veterinary Medical Association

**Published:** 1890-10

**Authors:** S. J. J. Harger

**Affiliations:** Secretary


### SOCIETY PROCEEDINGS.

*Pennsylvania State Veterinary Medical Association.*—The semi-annual meeting of the Pennsylvania State Veterinary Medical Association was held on September 2d, in the City Hotel, Lancaster. The president, Dr. Kooker, called the meeting to order, 10 A. M. On roll call the following members responded to their names: Drs. Kooker, Hart, Collins, Bradley, Weber, Hoskins, Gladfelter, T. B. Rayner, Harger, Custer, Magill, Foelker, Glass, Ridge, J. Curtis Michener, Huidekoper, James B. Rayner, Lusson, Bachman.

The following were present as visitors: B. F. Eshleman, J. J. Smith, R. H. Dreibelbis, C. O. Collins and Franklin M. Kain.

The reading of the minutes of the last meeting, with one correction, was approved. Dr. John J. Smith and P. K. Dreibelbis were favorably reported by the board of trustees and elected to membership. The name of A. Liautard, H.F.R.C.V.S., was offerred by Dr. Hoskins, and he was elected to honorary membership. Under the head of reports of committees, Dr. Hoskins, chairman of the Committee on Legislation, reported the decision in four cases tried under the Registration Act. These decisions not being of a favorable character, it was deemed best to have the bill amended at the next sesssion of the State legislature before prosecuting any further violation of the act. Dr Robert Gladfelter, corresponding secretary, gave a report covering forty of the counties in the State, showing in some of them a disposition to respect the requirements of the act; in others a continuation of registration. The report of committee, on motion, was adopted, and the committee continued with the view of preparing the necessary amendments for the coming session of the State legislature.

Dr. Huidekoper, chairman of the Committee on Intelligence and Education, made the following report:

REPORT OF COMMITTEE ON INTELLIGENCE AND EDUCATION.

During the last year there have not occurred any special matters of great importance which have been of direct interest to veterinary medicine, nor by which the latter can claim to have accomplished a marked stage in its history; nor has there been anything done by the members of the profession which has brought them very prominently before the world, or added much lustre to the craft.

However, although your committee cannot signal to you any one great victory to be proud of, they do believe that the large numbers of little matters which have been accomplished, in various ways tend to open the road for greater advances, and indicate a general and marked amelioration in the status of the profession, showing in it, and in the demands of the community, a desire for still further improvement.

The registration of veterinarians in the State, even with the defects in the Act, has had an excellent effect in establishing in the minds of the public the recognition of the responsibility attached to the position of veterinarian; and in binding the veterinarians together more as a body. The registration, even if it includes everyone who has any claim or pretense for calling himself a practitioner, at least forces him to put on record and within reach of the community, the information and data upon which he bases his assumption of his title. As we get older, the registration list will weed itself out of those names which should not have been on it; and the new generation, whose names are to be added to it, will prepare and qualify themselves before undertaking to enter as practitioners. The facilities for following the groundwork course of veterinary education in a school have not been such in the past as to admit of any large number of men availing themselves of them, but now they are so increased, and are placed within such easy access of all, that younger men will not be satisfied to trust in educating themselves; and the public will demand that the new practitioner, whom they call in for a case, shall give proof of his right to practice. Almost all of the veterinary schools of the country had larger classes last year than they have had in previous years; on their lists of students we find a notable increase of Pennsylvanians. A Pennsylvanian was awarded the highes honor last session for attaining the first place in studies in the graduating class of the American Veterinary College, New York. The classes in the veterinary schools are not only larger, but in most of the schools the courses of instruction have been extended, and the facilities for teaching have been improved. The American Veterinary College, New York, has added several chairs to its faculty, including that of sanitary medicine and veterinary jurisprudence, and has appointed Drs. Ryder, Bell and Huidekoper to its teaching staff. The course has been slightly lengthened; new buildings and a more extended course are in view. The New York College of Veterinary Surgeons has improved its buildings by a new museum and dissecting room. The University of Pennsylvania has added new rooms to its veterinary department, and has established State and city scholarships. The State of Ohio has appropriated a small sum for a veterinary building in connection with the University at Columbus.

The Chicago Veterinary College has enlarged its hospital, and erected a new laboratory, lecture room and dissecting room. The University of Minnesota has just established a veterinary department, which, presumably, replaces the Northwestern Veterinary College. The Montreal School has become a part of the McGill University, and the course has been improved by lectures on zoötechnics and canine medicine. The Ontario College has made additions to its buildings. All of these changes indicate a more prosperous condition of veterinary matters, the demand for a better veterinary education on the part of students, and an increase in the number of the latter. The Pennsylvania State Veterinary Medical Association has held large and instructive meetings, and the application for membership shows the interest which it is awakening. The Keystone Veterinary Medical Association (local association of Philadelphia and adjoining counties) has established its meetings in the building of the College of Physicians, of

Philadelphia, and so has obtained recognition as one of the medical societies, in common with the County Medical, the College of Surgery, the Obstetrical and Pathological Societies, etc. Local societies have been formed in the Northwestern portion of the State, taking in several neighboring counties of Pennsylvania, New York and Ohio, and a county veterinary society has been formed in Franklin County. In other States the veterinary societies are found in greater numbers, and doing more active work.

The decision of the United States Veterinary Medical Association, to hold its meeting this year in Chicago, can only be productive of great good in amalgamating the fellow-feeling of the veterinarians in the two sections of country, and in bringing before each the newer ideas of the other. It would be well for the various associations to have a closer affiliation than they now have. This would prevent faults in the constitution and by-laws of new societies, and would serve to correct errors which exist in older ones. Each county or local association should send one accredited delegate to the State meetings, who should be empowered to act for their associations in matters of business. Matters of ethics or questions of professional morals, which cannot be decided in a local meeting, should be referred to a State board. The State board should also have the power of hearing appeals from decisions by local societies. The State Associations should again be in harmonious relations with those of other States, and all should be tributary to the United States Veterinary Medical Association.

Your committee respectfully recommend :

I. That efforts be made to establish county veterinary associations throughout the State.

II. That where such county associations exist, with constitution and by-laws approved by the Pennsylvania State Veterinary Association, the president of the local association shall be *de facto* an advisory member of the Board of Trustees of the Pennsylvania State Association, without vote.

III. That in counties where such associations exist, no veterinarian shall be eligible for membership of the State association unless he is a member of the county association.

Your committee desire, further, to call attention to the frequent use of pictures and other excessive illumination on the bills and cards of veterinarians. While there is, apparently, no direct harm in the use of a horse's head, or a stallion rampant, a bull, a fowl or a whole barnyard on a bill head, and there seems precedent for it in the stationery of some of the veterinary schools, yet the code of ethics of most of the veterinary societies forbids its use, as tending to be carried to an extreme, and your committee recommend that a resolution be passed as follows :

*Resolved*, That the use of the pictures of animals on the stationery of members of this association be deprecated.

As a membership in this association is a recognition by a hundred prominent veterinarians throughout the State that such member is a reputable practitioner, and is the best guarantee which can be given to the public of his standing, and as the members are frequently called upon to recommend fellow-practitioners in other counties, your committee recommend that the officers of this association issue an association letter paper, having on one side a list of the officers and all the members of the association, by counties, with their addresses, and that such paper may be obtained from the secretary of the association, with the addition of the member's own heading, at cost.

Respectfully submitted,

RUSH S. HUIDEKOPER, *Chairman*,
H. G. PAGAT,
JAMES A. SALLADE, *Committee*.

The report was received, and it was ordered that 500 copies should be printed for distribution. The chairman on sanitary science and police being absent, reports from different sections of the State by various members were listened to. The subjects of glanders and contagious pleuro-pneumonia were discussed at some length, and the extremely meagre authority of the veterinarians over such cases was severely commented upon. The delegates to the New Jersey State Veterinary Medical Association, through Dr. Magill, reported that they had discharged their duties and were very hospitably entertained by the association. The president, under the amendment to the by-laws made the following additional appointments to the Sanitary Science and Police Committee :

Dr. S. K. Hoffman, Hamburg, Pa. ; Dr. J. W. Sallade, Pottsville ; Dr. George Magee, Uniontown ; Dr. J. L. Bradley, Mercersburg. Drs. J. Curtis, Michener and S. E. Weber were appointed a committee to draft suitable resolutions on the death of Dr. S. S. Moyer, and reported as follows :

*Resolved*, That we regret to learn of the death of our fellow-member and co-worker, Dr. Simon S. Moyer, and hereby extend our sympathy to the bereaved family.

*Resolved*, That we had learned to regard Dr. Moyer as a young man of great worth and integrity, ardently devoted to his chosen profession, and that our association loses a highly-respected member and the public a very useful citizen.

Communications from Drs. Birch, Stanton and Grove, regretting their inability to be present, were read by the corresponding secretary.

Under the head of essays, Dr. Glass read an article entitled "Cannabis Indica," as follows :

"*Cannabis Indica*, or cannabis sativa, is a drug that is comparatively little known or used by the veterinary profession. A brief description will assist us in the study of its actions. It is a native of India and the United States ; in the latter country only a small quantity is raised, and that of inferior quality. It is a small plant consisting of a bunch of bright green leaves and a flower stalk in the centre. The female plant is the one used. Its active principle is a resin, dark brown in color, of peculiar odor and a warm, pungent taste. The fluid extract is a dark brownish-green in color, a warm pungent taste and a peculiar fishy-like odor ; this smell is so characteristic that it is a drug easily remembered. The physiological action of this drug in large doses, especially in man, is very peculiar, one symptom being that the idea of time seems to be entirely lost, minutes seem hours, hours seem ages; of course, in the horse the recognition of these fancy flights is lost. In the administration of a series of ounce doses of the fluid extract, I first found a condition of stimulation and even exhilaration; the pulse increases to sixty, or even higher, the eye brightens, the pupil is said to dilate, but I could only find it clearly marked in one case that I experimented on; the intestinal murmur is slightly decreased, the animal presents an appearance of animation. This is followed by a condition of depression in which the pulse falls five to ten beats, the respirations are slower; the condition is one of overstimulation followed by reflex action. Therapeutically, it is probably the best drug in existence for spasmodic colic in the horse or dog; its effects are gradual and permanent. This is very clearly seen in a slow case of colic due to some irritant passing through the bowels. The spasms are slowly but surely relieved, the pulse is reduced in frequency. In the use of opium in a quick case due to flatulence, where the effect of a narcotic is prompt, the difference is not so marked, but in a slow case after repeated doses of opium the pulse becomes slow and weak, the animal begins to show that peculiar delirium of opium poisoning, walking around the stall in a dazed manner, the pupil contracted, the eye staring and glossy, the skin hot, dry, and the mouth dry and congested, presenting externally symptoms which are similar to enteritis. On recovery the animal is in a weak dazed condition for several days. With the fluid extract of cannabis indica these symptoms are not noticed; in some cases the after-depression may be slightly noticed, but very little, and I never saw a case in which there was any delirium following its administration. Another feature is that there is not the slightest tendency to constipation, so noticeable in the administration of frequent doses of opium. The dose as quoted in F. Dun, one drachm, is too small in cases of colic to rely on; there should be at least two drachm doses given, followed up at intervals of a half-hour by one drachm doses."

Dr. W. S Kooker called attention to the use of the drug by old Dr. Dadd in tetanus, and it was also recommended by others for epilepsy.

Dr. Weber reported a case of "Calculus of the Bladder" where he had performed lithotomy, and removed the stone, which was hollow, being filled with soft, partially organized matter. The incision was reported as healing over kindly.

The President appointed the following members as essayists for the next meeting: Prof. William L. Zuill, Dr. J. C. Michener, Dr. S. E. Weber, after which the meeting adjourned to meet in Philadelphia in March, 1891.

S. J. J. HARGER, *Secretary.*

